# Burn Care in Prisons: Ethical Dilemmas in Correctional Health Care

**DOI:** 10.1093/jbcr/irag139

**Published:** 2026-08-18

**Authors:** Joshua Khorsandi, Jason Mirharooni, Michael Kahen, Joshua Ahdout, Ava Niknejad, Demitri Franzoni, Joshua MacDavid

**Affiliations:** Department of Plastic and Reconstructive Surgery, Kirk Kerkorian School of Medicine at University of Nevada Las Vegas, Las Vegas, NV 89106, United States; Department of Medicine, Florida International University Herbert Wertheim College of Medicine, Miami, FL 33199, United States; Department of Plastic and Reconstructive Surgery, Kirk Kerkorian School of Medicine at University of Nevada Las Vegas, Las Vegas, NV 89106, United States; Department of Medicine, Touro University Nevada, Las Vegas, NV 89014, United States; Department of Plastic and Reconstructive Surgery, Kirk Kerkorian School of Medicine at University of Nevada Las Vegas, Las Vegas, NV 89106, United States; Department of Plastic and Reconstructive Surgery, University of Nevada Las Vegas, Las Vegas, NV 89106, United States; Department of Plastic and Reconstructive Surgery, University of Nevada Las Vegas, Las Vegas, NV 89106, United States

**Keywords:** burn injury, incarcerated patients, correctional health care, dual loyalty, health equity

## Abstract

Burn injuries among incarcerated individuals represent an underexplored intersection of medical ethics, correctional policy, and health equity. Although scholarship increasingly examines vulnerable groups such as refugees and undocumented patients, incarcerated patients with burn injuries remain largely absent. Dual loyalty—balancing duty to the patient with obligations to the institution or state—creates distinctive challenges that are intensified in burn care, where timely intervention, pain control, rehabilitation, and psychosocial support are critical but often constrained by security protocols and systemic neglect. We conducted a narrative review of PubMed, Embase, and Scopus to identify peer-reviewed articles, position statements, and ethical analyses addressing incarcerated healthcare, dual loyalty, and burn treatment (English-language, 2000-2025). A thematic synthesis identified recurring ethical tensions, clinical barriers, and potential frameworks for resolution. Findings highlight a major gap in both literature and professional discourse. Recurrent themes included restricted access to specialized burn centers, compromised informed consent under custodial oversight, inadequate rehabilitative services, and disproportionate suffering linked to institutional neglect. Dual loyalty emerges as a central driver of ethical conflict, often pitting patient dignity against correctional authority. Elevating incarcerated burn care within burn scholarship and advocacy is necessary to uphold standards of care. Explicit attention to dual loyalty can support reforms that preserve patient dignity while maintaining institutional safety, improve outcomes, and reaffirm professional ethical commitments.

## INTRODUCTION

Burn injury is a major global cause of morbidity and mortality; survivors often face functional, psychological, and social sequelae requiring resource-intensive care.^1^^,^^2^ Contemporary standards emphasize rapid access to burn centers, resuscitation/wound management, multimodal analgesia, early rehabilitation, and longitudinal psychosocial support.^3^ Yet access is unequal: incarcerated people have high burdens of chronic disease, mental illness, substance use disorders, and early-onset geriatric conditions in structurally constrained environments.^4^^,^^5^

Within correctional systems, ethical debate has emphasized infectious disease, mental health, palliative care, and opioid use disorder treatment, framed by equivalence of care.^6^^,^^7^ Acute injuries, including burns, remain neglected. Recent empirical work suggests incarcerated burn injuries are clinically and epidemiologically distinct—often smaller total body surface area burns, mechanisms such as boiling-water assault, and fewer operations than non-incarcerated patients^8^—and shaped by security logistics, transfer delays, and nonclinical constraints on care pathways.^9^

These dilemmas unfold in a carceral landscape marked by structural racism, overcrowding, and underinvestment.^10^ As Alsan and colleagues argue, constitutional protections against “deliberate indifference” coexist with weak oversight and cost-driven contracting, producing wide variation in correctional health standards.^5^ For burn clinicians, conflicts of dual loyalty are especially acute because obligations to patients may conflict with the expectations of correctional authorities.^11^

Comparable ethical debates arise in care for refugees, asylum seekers, and undocumented patients in detention or under deportation threat.^12–15^ This narrative review synthesizes literature on correctional health care, burn injury treatment, and ethics of care in custody and other constrained settings (including refugee and immigration detention), focusing on dual loyalty, limited burn-center access, consent under custodial oversight, analgesia and pain management disparities, and constraints on rehabilitation and psychosocial recovery. Figure 1 summarizes the correctional burn-care pathway and highlights where key ethical pressure points arise across transfer, treatment, and recovery.

**Figure 1:**
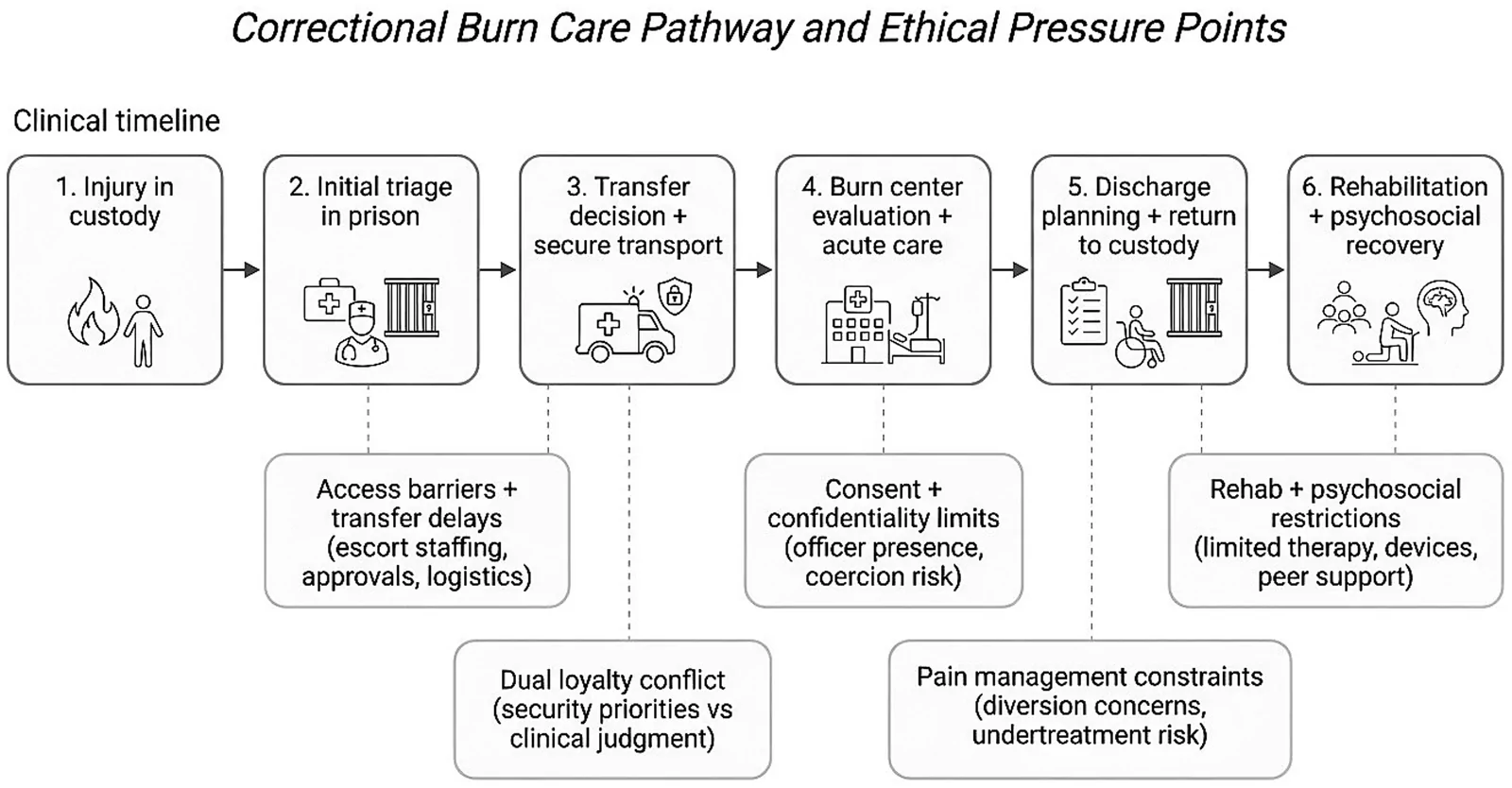
Correctional Burn Care Pathway and Ethical Pressure Points. Timeline of burn care in custody, showing where access delays, dual loyalty, consent limits, pain barriers, and rehab constraints arise.

## METHODS

This narrative literature review followed established guidance for narrative synthesis, prioritizing conceptual integration of heterogeneous evidence over quantitative aggregation. Peer-reviewed articles in English published between January 1, 2000 and May 31, 2025 were eligible for inclusion. Searches were conducted in PubMed, Embase, and Scopus using combinations of keywords and controlled vocabulary related to burns (“burn injury,” “burn center,” “burn rehabilitation”), incarceration (“prison,” “jail,” “correctional,” “carceral,” “incarcerated patients”), ethics (“dual loyalty,” “medical ethics,” “human rights,” “bioethics,” “equivalence of care”), and comparators (“refugees,” “asylum seekers,” “undocumented,” “immigration detention”).

The initial search strategy was iteratively refined by scanning reference lists of key articles in correctional health ethics, structural racism and health inequities, and burn rehabilitation. Conceptually relevant literature from closely related domains, such as palliative and end-of-life care in prisons, ethics of research with incarcerated populations, and clinical ethics analyses of care for people in immigration detention, was purposively sampled to enrich understanding of dual-loyalty conflicts and institutional constraints in custodial environments.

Studies were included if they: (1) addressed health care delivery, ethics, or health outcomes among incarcerated adults; (2) examined burn injuries in incarcerated populations or compared incarcerated and non-incarcerated patients; (3) analyzed dual loyalty or related conflicts in custodial or detention settings; or (4) explored access to, or quality of, burn care and rehabilitation in ways that could be meaningfully related to correctional contexts. Articles that focused exclusively on juvenile facilities, non-health topics, or noncustodial forensic settings were excluded. To enable comparative analysis, literature on health care for refugees, asylum seekers, and undocumented migrants was included when it addressed clinical ethics, dual loyalty, or structural barriers to care in detention or enforcement settings.

Titles and abstracts were screened for relevance, followed by full-text review. Data were extracted narratively, with attention to the nature of the population, setting, clinical focus, ethical issues identified, and any conceptual or policy frameworks proposed. In total, 48 peer-reviewed articles met inclusion criteria and informed the final narrative synthesis. Thematic synthesis was then used to identify recurrent domains of ethical tension, including dual loyalty, access to burn centers, informed consent and decision-making, pain management, and rehabilitation and psychosocial recovery. The synthesis also examined how structural determinants, such as mass incarceration, structural racism, and health system financing, shaped these domains. The aim was not to provide an exhaustive catalogue of all relevant publications, but to construct a conceptually rich account of ethical dilemmas in correctional burn care grounded in contemporary empirical and normative scholarship.

## RESULTS

### Limited empirical evidence on burns in incarcerated populations

Only a small number of peer-reviewed studies directly examine burn injuries in incarcerated individuals, but these studies provide important clinical data on frequency, mechanism, burn extent, management, and outcomes. In a 10-year retrospective series from a regional burn center in the United Kingdom, Jawad et al. identified 18 burn referrals from 3 prisons between 2007 and 2017, with 44% of referrals occurring in 2017. Most injuries were scald burns (94%), more than half were assault-related (56%), and injuries commonly involved hot-water kettles targeting the face and trunk. The mean burn size was 2.89% TBSA, with a range of <1% to 8%; 80% of patients received first aid, 72.2% were admitted to the hospital for an average of 4 days, and all burns were managed nonoperatively with dressings and an average of 2 outpatient reviews.^16^

Nosanov et al. queried the National Burn Repository for adult patients with burn injuries discharged into custody between 2002 and 2011, identifying 809 patients discharged to jail, representing 0.7% of eligible adult patients in the dataset. Of these, 283 patients (35.0%) sustained their injuries while in custody. Patients were predominantly male (86.2%) and White (52.3%), with a median age of 35.7 years. Compared with non-incarcerated patients, incarcerated patients had significantly smaller burns (2.0% vs 3.8%), were less likely to undergo an operation, and had comparable hospital lengths of stay. The authors concluded that although incarcerated patients with burn injuries sustained smaller injuries and received fewer operations, they remained hospitalized for similar durations as non-incarcerated patients.^8^

In the Birmingham Burns Centre experience, Rafie et al. identified 68 prisoners with burn injuries from 2010 to 2021. The median age was 28.5 years, the median burn size was 3% TBSA, and nearly all patients were male (98.5%). Most patients required hospital admission (75%). Scalds were the most common injury type (77.9%), and assault was the most frequent cause of burn injury (63.2%). Eighteen patients (26.5%) underwent a surgical procedure, and 2 deaths were reported. Follow-up was also frequently disrupted: among patients for whom follow-up was planned, 22% attended no appointments and an additional 49% missed at least 1 appointment.^9^

Taken together, these studies suggest that incarcerated patients with burn injuries represent a small but clinically distinct burn population, often characterized by relatively small TBSA burns, scald mechanisms, assault-related injury, logistical barriers to care, and disrupted outpatient follow-up. However, the literature provides minimal detail on patient experiences of pain, decision-making, or rehabilitation, and almost no data on long-term functional, social, or psychological outcomes. In stark contrast, numerous studies of burn survivors in the general population document complex trajectories of physical recovery,^17^ return to work,^18^^,^^19^ and social reintegration,^17^^,^^20^ as well as high rates of depression, anxiety, and posttraumatic stress.^21–23^ The near absence of analogous data for incarcerated patients signals a major empirical and ethical blind spot. Table 1 summarizes the limited studies that directly evaluated burn injuries in incarcerated patients. Furthermore, Figure 2 summarizes where evidence is strong versus missing across the key ethical domains in correctional burn care.

**Table 1:** Key Studies on Incarcerated Patients With Burn Injuries

Study	Key takeaway	Gap highlighted	References
UK regional burn center review	Prison burns often involved scald/assault; transfers/logistics were major burdens.	Little detail on pain, consent, rehab, or long-term outcomes.	^16^
National Burn Repository analysis	Smaller burns and fewer operations in incarcerated patients; similar LOS	Suggested nonclinical constraints may affect care decisions.	^8^
“Patient or Prisoner?” single-center series	Transport delays and custodial rules affected timing of surgery/rehab.	No systematic patient-experience or recovery outcomes reported	^9^

Abbreviations: LOS, length of stay; rehab, rehabilitation; UK, United Kingdom.

**Figure 2:**
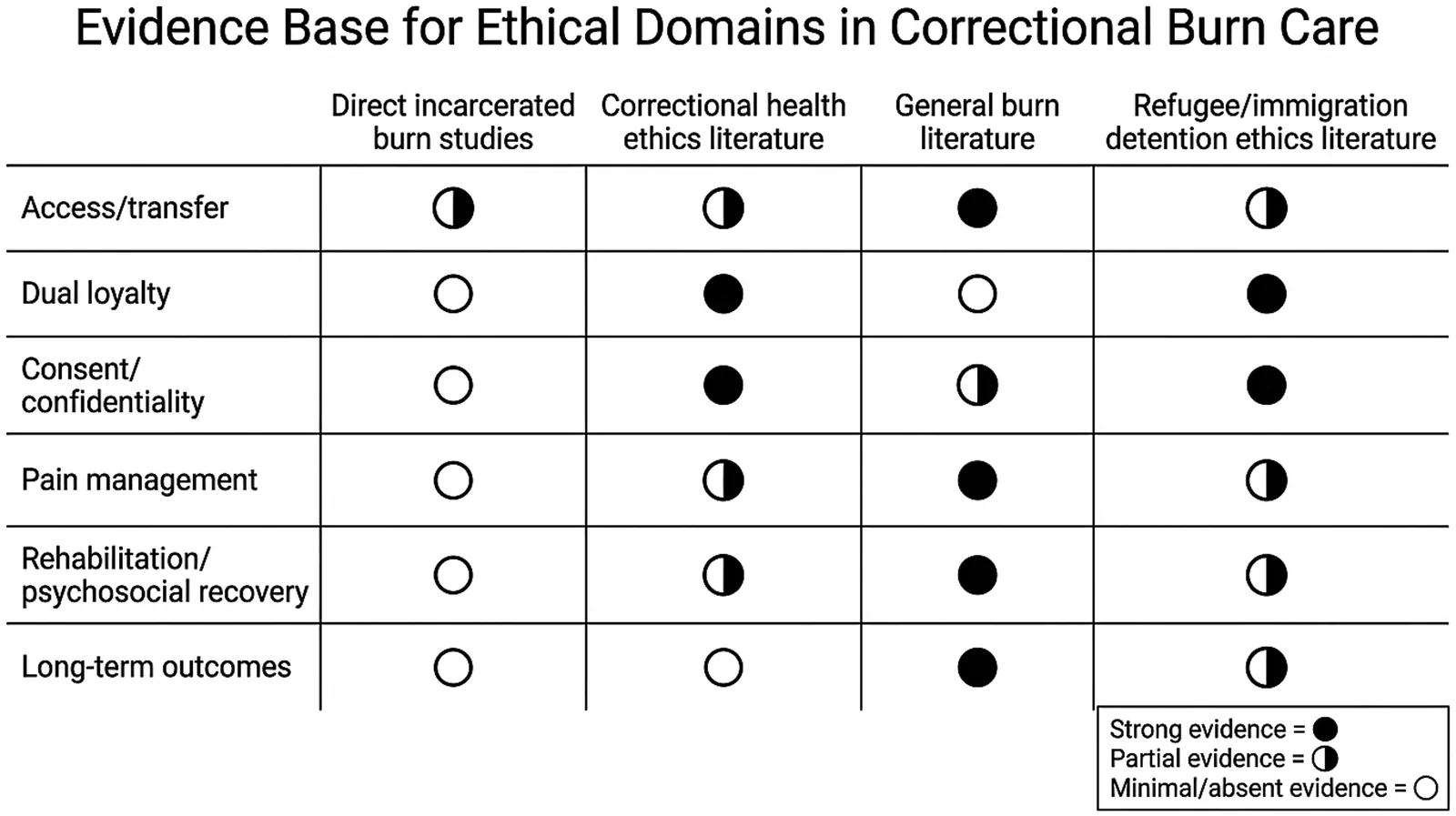
Evidence Gaps in Correctional Burn Care Ethics. Matrix showing how limited direct prison-burn evidence is across access, dual loyalty, consent, pain, rehab, and long-term outcomes.

### Barriers to timely access to specialist burn centers

Across the broader burn literature, timely access to specialist centers is repeatedly associated with improved survival, functional recovery, and quality of life.^24–26^ Research letters and registry analyses in the United States show substantial geographic inequities in burn-center access, particularly by region and socioeconomic status.^24^ Because timely specialist burn care, multidisciplinary management, and rehabilitation are central to morbidity, mortality, functional recovery, and quality of life, these access gaps may have important implications for outcomes in non-incarcerated populations.^2^^,^^24–26^ These access barriers are amplified for incarcerated patients, for whom transfer requires coordination of custodial staff, secure transport, and often judicial or administrative approvals.^16^

Correctional health and public health scholarship further emphasize how mass incarceration, chronic understaffing, and fragmented governance create structural barriers to accessing nonemergency hospital care. Alsan and colleagues describe wide variation in contracting models, performance metrics, and oversight mechanisms, noting that cost-containment incentives can discourage external referrals or specialist consultations.^5^ Editorials and empirical studies on prison healthcare argue that poor prison conditions, including overcrowding and security-driven movement restrictions, directly compromise the ability of clinicians to arrange timely transfers and follow-up appointments for serious health conditions.^27^^,^^28^ Observational work in other specialties, such as ophthalmology, reaches similar conclusions, documenting missed appointments and disruptions in care when security arrangements take precedence over clinical need.^29^

In addition to institutional transfer delays, incarcerated patients with burn injuries may also experience patient-mediated delays in presentation. Within correctional settings, individuals may be reluctant to disclose injuries, particularly those arising from interpersonal or gang-related violence, due to fear of retaliation or being labeled a “snitch.” As a result, some patients present days after injury, often with infection or worsening wounds. These delays complicate accurate assessment of injury severity and timing, hinder timely intervention, and reflect an underrecognized interaction between carceral social dynamics and clinical outcomes.

### Case-based examples of correctional burn and fire-related care

Published case-based examples illustrate how burn and fire-related injuries in custody can create clinically and ethically complex situations. Williams and Giles described a prisoner who sustained superficial partial-thickness burns to the back and shoulder after being assaulted with scalding water within 48 h of arriving at prison.^30^ Referral to a specialist burns service led to an outreach nurse assessment, development of a care plan within the prison health center, and subsequent recognition of anxiety, fear, low mood, and the need for referral to a consultant psychologist.^31^ Fortin et al. reported a 23-year-old prisoner found in cardiorespiratory arrest after approximately 30 min of smoke exposure from a burning mattress during an apparent suicide attempt; elevated cyanide and lactate levels supported the diagnosis of cyanide poisoning, and timely hydroxocobalamin administration contributed to full neurologic recovery.^31^ Together, these cases demonstrate that correctional burn and fire-related care may involve not only wound management or emergency resuscitation but also assault, self-harm, mental health needs, specialist access, privacy, follow-up, and institutional responsibility.

In the scald-assault case, the ethical and clinical challenges were navigated through collaboration between prison health services and a specialist burn outreach team, allowing burn assessment and wound-care planning to occur within the prison setting while also identifying psychological distress requiring referral. In the smoke-inhalation case, clinicians navigated the emergency by recognizing cyanide poisoning as a possible cause of cardiorespiratory arrest after prison fire exposure, initiating resuscitation and hydroxocobalamin treatment, and providing hospital-based critical care support. These published cases suggest that correctional burn and fire-related care requires both immediate clinical response and systems-level coordination across prison staff, specialist burn services, emergency care, mental health care, and follow-up planning.

Although these published cases help illustrate the clinical complexity of burn and fire-related injuries in custody, the existing literature offers limited practical guidance for navigating such scenarios. Available studies and ethics analyses identify recurring concerns, including delayed access to specialty care, dual loyalty, compromised privacy, barriers to informed consent, undertreated pain, and disrupted rehabilitation, but they rarely provide concrete case-level guidance for clinicians. In particular, the literature does not clearly define how burn teams should balance urgent transfer needs with security restrictions, how private consent discussions should be protected when correctional officers are present, how burn-related pain should be managed when diversion concerns exist, or how rehabilitation and follow-up should be ensured after return to prison. This gap limits the ability of clinicians and institutions to move from recognizing ethical problems to implementing consistent safeguards.

### Dual loyalty as a central ethical tension

Dual loyalty was the most consistently described ethical theme across the correctional health literature. Sources defined dual loyalty as conflict between clinicians’ duties to patients and obligations to custodial authorities, with recurring examples including security-driven limits on clinical independence, disclosure of health information to correctional staff, and pressure to prioritize institutional order over patient welfare.^11^^,^^32^^,^^33^

Dual loyalty conflicts are particularly salient in burn care, where clinical decisions about transfer, debridement, grafting, and intensive rehabilitation have profound implications for pain, function, and long-term outcomes. The limited burn literature on incarcerated patients hints at situations in which bed availability, escort staffing, or security concerns shaped whether and when patients received operative care or were transferred back to the correctional facility, sometimes earlier than would be typical for non-incarcerated patients.^9^^,^^16^ Ethical analyses of prison healthcare more broadly emphasize that such decisions are rarely neutral: when institutional priorities are allowed to override clinical judgment, the principle of equivalence of care is undermined and the risk of inhumane treatment increases.^6^^,^^7^

### Informed consent and decision-making under custodial oversight

The ethics of informed consent and surrogate decision-making for incarcerated patients have received growing attention. Eichelberger et al. argue that hospitalized incarcerated patients should be treated as any other patient, with confidentiality, professional independence, and equivalence of care upheld even when security staff are present; they warn that allowing wardens or correctional officers to influence treatment decisions violates basic ethical standards.^7^ An ethics analysis in the *AMA Journal of Ethics* similarly maintains that carceral representatives should not act as surrogate decision-makers for patients who retain capacity, and that when capacity is impaired, standard state law processes for surrogate designation must be followed rather than defaulting to correctional administrators.^34^ Related literature in palliative care and surgical research ethics similarly emphasizes that custodial policies can constrain communication, voluntariness, privacy, and continuity of care for incarcerated patients.^35^^,^^36^

For burn care, these insights imply that informed consent discussions about debridement, grafting, reconstructive procedures, or high-risk sedation must occur in environments that protect privacy and minimize coercive influence from security staff. However, empirical descriptions of how consent is actually obtained for incarcerated patients with burn injuries are virtually absent. The lack of data contrasts sharply with detailed accounts of how trauma, language barriers, and legal precarity complicate consent in refugee and asylum contexts.^13^^,^^15^

### Disparities in analgesia and pain management

Pain is a central feature of burn injury and rehabilitation. Outside correctional settings, systematic reviews document complex interactions between physical and psychological pain, with unrelieved pain impeding wound care participation, sleep, mood, and social recovery.^17^^,^^37^ Broader pain literature reveals persistent racial and ethnic disparities in analgesia provision, including lower odds of opioid or ketamine administration for minoritized patients in prehospital and emergency settings.^38^^,^^39^ These patterns intersect with structural racism and mass incarceration, given the overrepresentation of Black, Indigenous, and other people of color in prisons.^10^

Within correctional health, qualitative and policy analyses describe chronic undertreatment of pain due to concerns about diversion, malingering, and substance use, with consequent reliance on non-opioid regimens and abrupt deprescribing of long-standing analgesics when individuals enter custody.^40^ Commentaries on carceral pain highlight ethical tensions between diversion control and the obligation to relieve suffering, arguing that equivalence of care requires robust, multimodal pain strategies rather than categorical restrictions on certain drug classes. Despite this, specific guidance on burn-related pain in incarcerated populations is lacking.

### Rehabilitation, psychosocial recovery, and institutional constraints

Rehabilitation following burn injury encompasses physical therapy, occupational therapy, scar management, vocational support, and psychosocial interventions. Previous studies show that structured exercise programs and multidisciplinary rehabilitation improve strength, aerobic capacity, and quality of life,^3^^,^^26^ and facilitate return to work and social roles.^18^^,^^19^ Psychosocial reviews emphasize that community reintegration,^41^ vocational participation,^42^ and peer support^43^^,^^44^ are critical determinants of long-term wellbeing. Prison-focused literature describes fragmented rehabilitative and psychosocial services, limited specialized staff, high burdens of functional impairment, and security restrictions that can limit access to assistive devices, group therapies, and community-based programs.^4^^,^^45–47^

No identified study systematically evaluated burn rehabilitation or psychosocial recovery among incarcerated patients. Extrapolating from both burn and prison rehabilitation literature suggests that incarcerated burn survivors are likely to experience compounded barriers, with restricted access to intensive physical therapy, peer support programs, and vocational rehabilitation, alongside ongoing exposure to stressors such as overcrowding, violence, and solitary confinement.

### Comparisons with refugees, asylum seekers, and undocumented patients

Several strands of literature examine health care for refugees, asylum seekers, and undocumented patients, particularly in detention or under immigration enforcement. Systematic reviews and qualitative studies show that refugees frequently encounter fragmented care, language barriers, uncertainty about entitlements, and distrust of authorities, all of which impede timely access to specialized services and continuity of care.^48^ Ethical analyses of offshore and onshore detention facilities emphasize dual-loyalty conflicts, with health professionals pressured to prioritize deterrence or deportation over patient welfare.^13^^,^^14^ Commentaries on health care for undocumented immigrants similarly highlight conflicts between clinicians’ duties of confidentiality and collaboration with immigration enforcement systems.

These populations share with incarcerated people exposure to coercive state systems, restricted movement, and fear of authority. Compared with the prison-burn literature, however, refugee and undocumented-care scholarship offers more developed ethical frameworks, including human rights-based and trauma-informed approaches.

## DISCUSSION

### Incarcerated burn patients as a neglected ethical frontier

The limited literature on burn care in correctional settings indicates that incarcerated patients with burn injuries remain an ethical blind spot within burn care scholarship. Rather than repeating the descriptive findings above, the key implication is that current evidence is not sufficient to evaluate whether transfer timing, operative decisions, pain control, rehabilitation intensity, and long-term outcomes meet community standards. This imbalance matters clinically and ethically. In the absence of systematic data on transfer timing, operative management, pain control, rehabilitation intensity, and long-term outcomes, care can default to security-centered policies or ad hoc decisions that are difficult to benchmark against community standards. This invisibility conflicts with equivalence of care and commitments to reduce carceral health inequities.^6^^,^^7^

### Ethical complexity of consent and decision-making

In custodial settings, autonomy requires more than signed consent; it requires protection from coercion and undue influence.^11^^,^^34^ Burn decisions (surgery, amputation, reconstruction, and high-risk sedation) are value-laden and occur amid pain and fear; officer presence, or beliefs that refusal could affect parole or living conditions, can compromise voluntariness. Detention literature shows how power imbalances can distort consent even when documentation seems adequate.^13^ A human rights-based approach would treat incarcerated patients with burn injuries as rights-holders by ensuring private spaces, limiting officer presence during sensitive discussions, explicitly separating treatment choices from punishment or parole consequences, and using independent advocates where available. Ethics consultation can assist when institutional policies conflict with patient preferences.

### Structural inequities and disparities in analgesia and rehabilitation

Structural racism scholarship emphasizes that inequities are produced by policies and institutions.^10^ Communities most likely to be incarcerated are also at higher risk for burn injury and undertreated pain; disparities in emergency and prehospital analgesia (including opioids and ketamine) are documented for racially and ethnically minoritized patients.^38^^,^^39^ In prison, diversion concerns and institutional control further constrain pain care, including deprescribing on entry, limited non-pharmacologic options, and patients being disbelieved or labelled manipulative.^40^ While diversion risk justifies careful titration within multimodal plans, blanket restrictions without individualized assessment are ethically problematic and may worsen suffering during dressing changes, mobilization, and rehabilitation.^37^

Rehabilitation inequities are similar. Burn rehabilitation evidence supports intensive multidisciplinary programs and reintegration efforts for return to work or community participation,^3^^,^^43^ but correctional facilities often restrict movement and social contact. Without adaptations (in-prison physiotherapy capacity, tele-rehabilitation links, and community partnerships), rehabilitation will likely be truncated. Addressing these inequities will likely require institution-level policies that prioritize continuity of rehabilitation, protected access to multidisciplinary services, and individualized pain-management strategies that are not overridden solely by security concerns. Table 2 links the article’s main ethical dilemmas to practical safeguards discussed in the Discussion.

**Table 2:** Core Ethical Dilemmas and Practical Safeguards

Ethical issue	How it shows up	Practical safeguard	References
Dual loyalty	Security priorities can shape transfer timing, surgery, and discharge back to prison	Protect clinical independence; avoid security-based overrides.	^9^ ^,^ ^11^ ^,^ ^16^
Consent/confidentiality	Officer presence and institutional influence can undermine voluntariness.	Private consent when possible; limit officer role in decisions.	^7^ ^,^ ^11^ ^,^ ^32^
Pain + rehab inequities	Undertreated pain and restricted rehab/psychosocial supports.	Individualized multimodal pain plans; adapt rehab access (eg, tele-rehab/in-prison capacity).	^3^ ^,^ ^38^ ^,^ ^45^

Abbreviations: rehab, rehabilitation; tele-rehab, tele-rehabilitation.

### Lessons from other detained populations and pathways forward

Refugee and literature on care for undocumented patients highlights fragmented access, mistrust, and ethical risks when clinicians are pressured into enforcement-aligned roles.^13^ For burn care, this supports professional guidance and collective advocacy: organizations could issue position statements affirming primary loyalty to patient welfare and rejecting security-based overrides of pain management, consent, and rehabilitation, informed by trauma-informed, rights-based frameworks.

Improvement frameworks include: (1) moving equivalence from access to equivalence of outcomes via routine comparison of transfer times, operative rates, pain scores, rehabilitation intensity, and long-term outcomes^11^; (2) a public-health approach framing incarceration as a health crisis and supporting decarceration/medical parole and, when necessary, compassionate release if prisons cannot support required care^5^; and (3) structural competency training and systems work (morbidity and mortality [M&M] review of incarcerated cases, correctional burn pathways, and registry inclusion of incarceration status) plus burn-relevant correctional pain guidance balancing diversion control with relief of suffering.^10^ Finally, priorities for empirical work include patient-experience studies, comparative cohorts, and implementation evaluations of outreach or tele-rehab models; the “Burns Behind Bars” approach suggests feasibility but requires rigorous ethical evaluation to ensure it advances care rather than normalizing injustice.^16^

To make these recommendations more actionable, we developed a practical correctional burn-care pathway that identifies key stages of care where inequities may arise and pairs each stage with a corresponding safeguard or institutional response (Figure 3). This pathway translates the ethical and structural concerns identified in this review into concrete clinical and systems-level intervention points.

**Figure 3:**
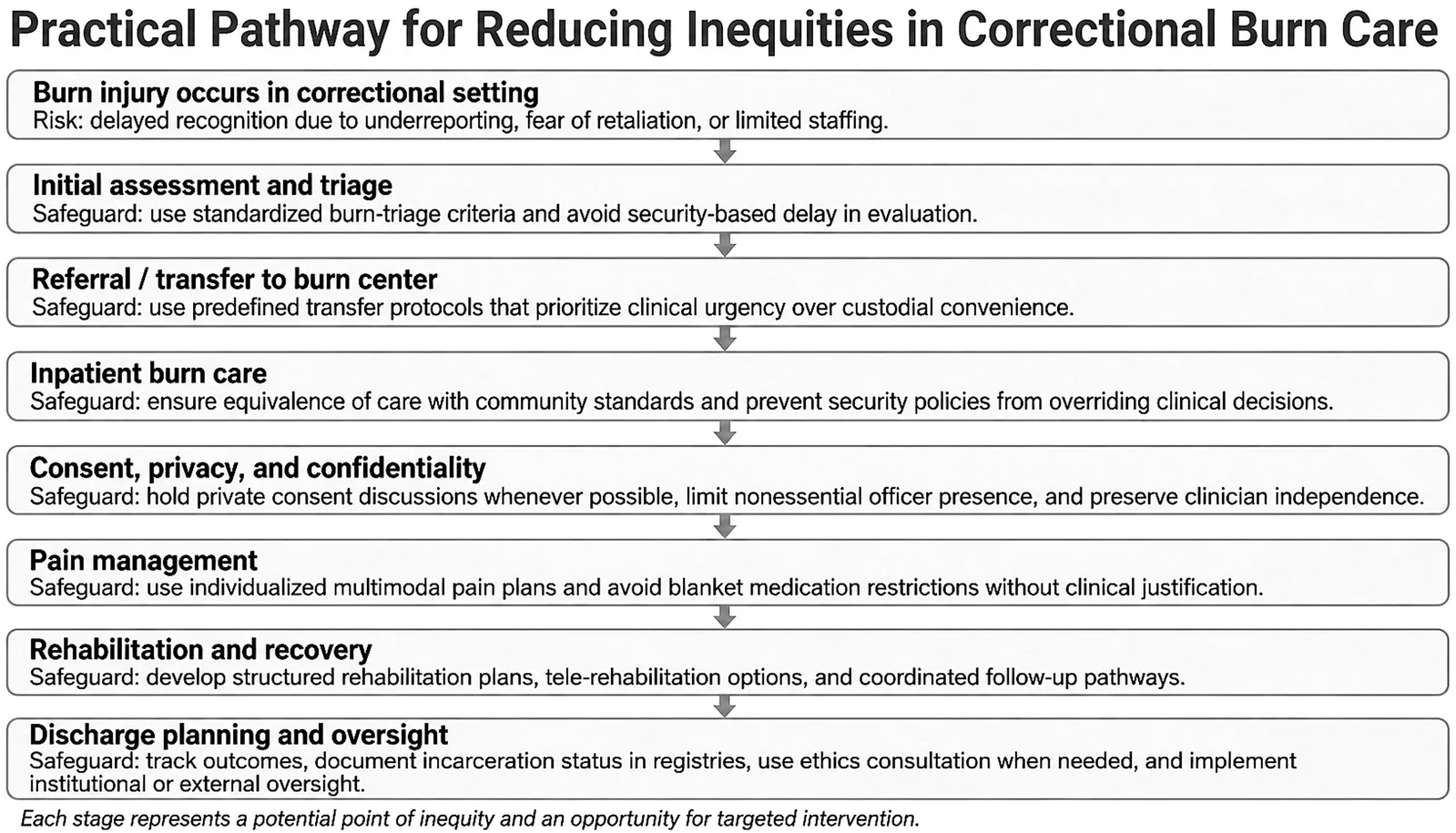
Practical Pathway for Reducing Inequities in Correctional Burn Care. This figure summarizes key stages in the care of incarcerated patients with burn injuries and highlights practical safeguards that may reduce inequities at each stage. Shown are points of potential inequity from injury recognition through triage, transfer, inpatient management, consent, pain control, rehabilitation, and discharge planning, together with corresponding institutional and clinical interventions.

### Conclusion

Incarcerated patients with burn injuries exist at a neglected intersection of trauma, structural inequity, and institutional control. This narrative review demonstrates that, despite isolated empirical studies, burn scholarship has largely failed to address the ethical and structural complexities of delivering specialized burn care, adequate analgesia, rehabilitation, and psychosocial support within carceral environments. Existing correctional health literature highlights recurring problems of dual loyalty, limited oversight, delayed specialty access, compromised confidentiality, and failure to achieve equivalence of care. At the same time, scholarship involving refugees, asylum seekers, and undocumented patients illustrates how coercive systems can distort clinical relationships, undermine autonomy, and compromise patient-centered care. Together, these literatures suggest that barriers to specialist access, constrained decision-making, undertreated pain, and disrupted rehabilitation are not secondary complications of incarceration, but central ethical challenges in correctional burn care.

Addressing these inequities will require coordinated institutional, clinical, and policy reforms that move beyond general calls for prison health improvement. Future ethical and clinical guidance for correctional burn care should establish clear standards for timely burn-center referral and transfer, protected informed-consent discussions, confidentiality during clinical encounters, individualized multimodal pain management, continuity of rehabilitation services, discharge planning, and long-term follow-up after return to custody. Safeguards are also needed to limit security-based interference with clinical decision-making and to ensure access to ethics consultation or external review when institutional priorities conflict with patient welfare. Routine documentation of incarceration status in burn registries and outcomes research should further support accountability and quality improvement efforts.

## Notes

Author contributions: Joshua Khorsandi (Writing—original draft [lead]), Jason Mirharooni (Writing—review & editing [equal]), Michael Kahen (Methodology [equal]), Joshua Ahdout (Data curation [equal]), Ava Niknejad (Investigation [supporting]), Demitri Franzoni (Supervision [lead]), and Joshua MacDavid (Supervision [supporting])

Funding: This research received no external funding.
